# Receptor Tyrosine Kinases: Molecular Switches Regulating CNS Axon Regeneration

**DOI:** 10.1155/2012/361721

**Published:** 2012-07-16

**Authors:** Vasanthy Vigneswara, Sarina Kundi, Zubair Ahmed

**Affiliations:** Neuropharmacology and Neurobiology Section, School of Clinical and Experimental Medicine, College of Medical and Dental Sciences, University of Birmingham, Edgbaston, Birmingham B15 2TT, UK

## Abstract

The poor or lack of injured adult central nervous system (CNS) axon regeneration results in devastating consequences and poor functional recovery. The interplay between the intrinsic and extrinsic factors contributes to robust inhibition of axon regeneration of injured CNS neurons. The insufficient or lack of trophic support for injured neurons is considered as one of the major obstacles contributing to their failure to survive and regrow their axons after injury. In the CNS, many of the signalling pathways associated with neuronal survival and axon regeneration are regulated by several classes of receptor tyrosine kinases (RTK) that respond to a variety of ligands. This paper highlights and summarises the most relevant recent findings pertinent to different classes of the RTK family of molecules, with a particular focus on elucidating their role in CNS axon regeneration.

## 1. Introduction

In the mammalian central nervous system (CNS), the failure of spontaneous regeneration of injured axons leads to devastating consequences and poor functional recovery. Severe injuries to CNS axons not only damage plasticity of synapses but also provoke complex degenerative cascades, leading to glial and neuronal apoptosis. The vast majority of injured CNS neurons progressively fails to regenerate beyond the lesion site to reestablish functional synaptic transmission and only a small number of axons show compensatory sprouting, resulting in poor functional recovery [1–5]. Lack or insufficient trophic support is one of the major determinants attributed to the failure of adult CNS axon regeneration. Growth factors that act both on neurons and glia, mediate a variety of physiological functions from early embryonic to the adult state, including synaptic plasticity, cell survival, and death in the CNS [6–10]. Hence, trophic factors and their corresponding receptor-mediated signalling pathways involved in neuronal survival and axon regeneration have been subjected to considerable attention. Many of these studies have been aimed at developing potential therapeutic interventions for the treatment of peripheral nervous system (PNS) and CNS injuries and certain neurodegenerative disorders like Parkinson's and Alzheimer's diseases. 

## 2. Mechanisms behind the Failure of CNS Axon Regeneration 

In general, functional axon regeneration is a multifactorial process; a myriad of molecules and a combination of signalling pathways are often involved. Two important prerequisites are essential for successful regeneration. Firstly, the injured neurons must be competent to survive after injury, since the replacement of compromised cells is a critical step in the healing process, and having the intrinsic ability to reexpress growth-promoting genes is necessary to stimulate axon regeneration. Secondly, there should be a permissive environment to support spontaneous axon growth and facilitate reinnervation of their target tissues [11, 12]. In contrast to CNS, injured axons in the PNS have the ability to regenerate and reinnervate their target tissues and thereby restore lost sensory and motor functions. The strikingly different responses of the CNS and PNS to injury and the molecular and cellular changes at the lesion sites are challenging issues to overcome in the treatment of severe CNS injuries caused by spinal cord trauma and stroke [2, 11, 13–15].

It is widely believed that most injured adult CNS neurons are intrinsically incapable of axon regeneration [11, 15, 16]. Increasing evidence emphasise that the inability of injured CNS neurons to regenerate is not entirely associated with their intrinsic deficits, but rather attributed to the generation of an inhibitory environment in the CNS. After injury, severed axons retain, at least in part, the regenerative capacity to form functionally active growth cones and produce axon extension over long distances in a permissive environment, rather than completely fail to regrow [4, 17–19]. The competence of injured neurons to regenerate in the presence of a permissive environment is also restricted to certain neuronal populations that show a varied degree of regenerative responses to similar environmental manipulations, which facilitate regeneration [12, 20–22]. 

The insufficient growth potential of CNS neurons results from the failure of the transition from the normally “transmitting” to the “growth mode” after injury, mainly due to their multiple collateral axons which remain connected to their various target tissues (e.g., long axon tracts in the spinal cord [23]). By contrast, PN injuries trigger robust reexpression of growth-promoting genes in injured neurons to produce a variety of neurotrophins and other growth associated proteins. For example, growth associated protein-43 (GAP-43) and CAP-23 are highly upregulated and correlate with the conversion of neurons to a growth activated state that facilitates survival and subsequent axon regeneration [24–28]. 

On the basis of several studies, the lack of axon regeneration of mature CNS neurons is ultimately due to a paucity of growth promoting cues, and especially the availability of growth promoting factors and their heightened susceptibility to a plethora of axon-growth inhibitory ligands. These include central myelin/oligodendrocyte-derived Nogo, myelin associated glycoprotein (MAG), and oligodendrocyte myelin glycoprotein (OMgp), the astrocyte-rich glial scar-derived inhibitory molecules such as chondroitin sulphate proteoglycans (CSPG) and tenascin that promote growth cone collapse and slow Wallerian degeneration with poor remyelination after injury [14, 23, 29, 30]. 

Indeed, axonal growth determinants in the CNS are complicated and the existing challenges to overcome these inhibitory cues and obtain complete functional recovery are substantial. To date, there remains a controversy regarding how injured neurons respond to these intrinsic and extrinsic cues to provoke the cascade of signalling pathways that disrupt or abort axon regeneration after injury. Several studies indicate that optimization of axon regeneration is a counterbalance between the intrinsic growth promoting ability of severed neurons and the growth inhibitory signals from the nonpermissive CNS environment [2, 29, 31]. During development, axon elongation is sequentially orchestrated by a series of signalling pathways to provoke necessary events both intrinsically and extrinsically in the environment of growing neuronal axons. This ensures accurate and rapid axon growth by an array of guidance cues towards their targets [9, 32, 33]. On the basis of all of these studies, manipulation of a single factor of intrinsic or extrinsic cues of a particular neuronal population may be sufficient to activate the Rho family GTPase intracellular signalling pathway, leading to actin depolymerisation in axon growth cones and preventing axon regeneration. Despite the fact that injured neuronal cells are exposed to a variety of both intrinsic and extrinsic stimuli that activate corresponding signalling pathways to elicit appropriate responses, how these signalling networks are interconnected to arrest axon regeneration is not well understood. Many of the signalling pathways associated with neuronal survival and axon regeneration are regulated by several classes of RTK in response to various types of ligands. Therefore, the intention of this paper is to provide an overview of the roles of some of the most relevant RTKs on CNS axon regeneration and explore their pharmacological properties.

## 3. RTK Family Members and Their Impact on CNS Axon Regeneration

**Figure 1:**
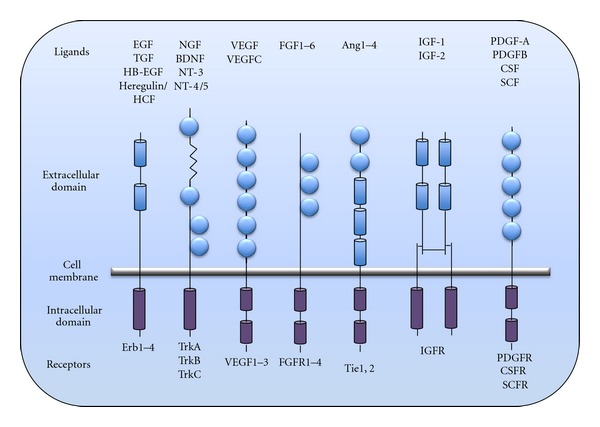
Different classes of RTK. RTK share tyrosine kinase domains in the intracellular portion while the extracellular portion contains cysteine repeat regions and single cysteine residues (circles). RTK family has a variety of ligands and receptors.

RTK are high affinity, cell surface receptors for many polypeptide growth factors, cytokines, and hormones. RTK-mediated signals play pivotal and diverse roles in the regulation of various physiological functions, ranging from cell proliferation, differentiation, cell adhesion, cell migration, survival, and apoptosis [34, 35]. In the human genome alone, 58 RTK are encoded and categorized into 20 subfamilies. These include nerve growth factor receptors (NGFR), tropomyosin-receptor-kinases (Trk) family receptors, epidermal growth factor receptors (EGFRs), fibroblast growth factor receptors (FGFR), glial cell-derived neurotrophic factor receptor (GFR), and the insulin and insulin-like growth factor receptor (IR and IGFR) (Figure 1) [36, 37]. The identification of several new members of RTK provides unique insights into their broader specificity and largely overlapping signal transductions, coupled with their multiple roles in the nervous systems.

### 3.1. RTK Structure and Signal Transduction

**Figure 2:**
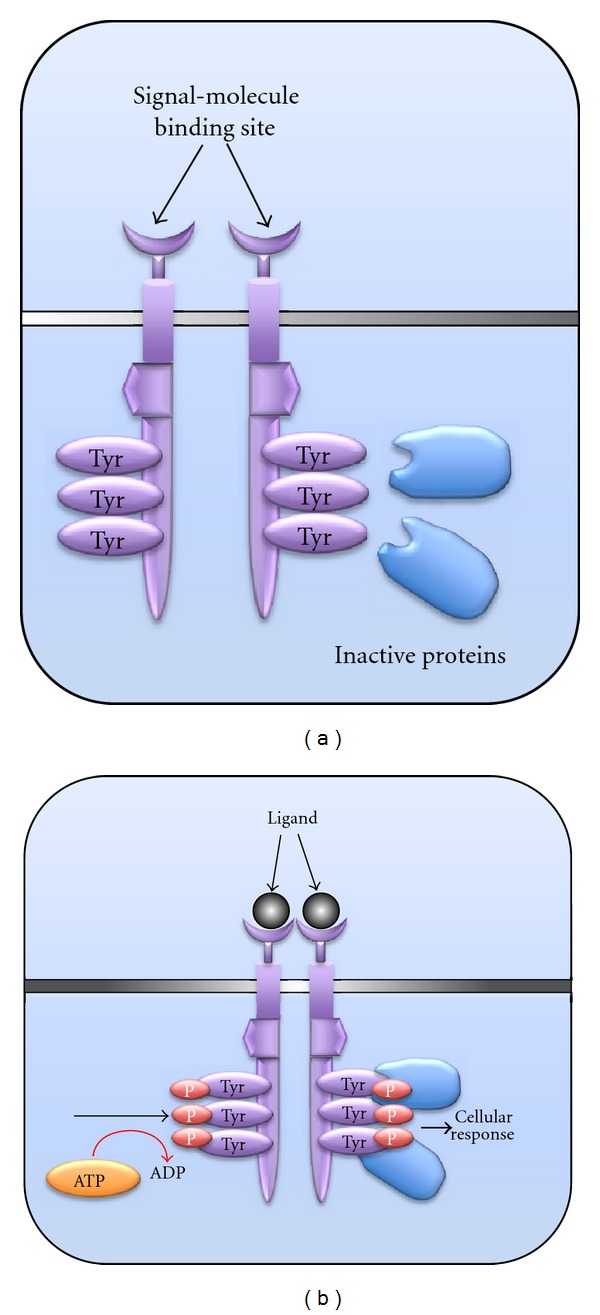
Activation of RTK signal transduction. (a) Inactive RTK are monomeric but after ligand binding dimerization of the extracellular domain occurs (b) and since cytoplasmic domains are juxtaposed, phosphorylation of tyrosine residues (ovals with Tyr labels) is facilitated. Phosphorylation allows inactive proteins to interact with the tyrosine residues and elicit appropriate cellular responses. Adapted from [50] Hubbard, 2004.

RTK contain four main domains: the extracellular ligand binding domain; the intracellular/cytoplasmic highly conserved catalytic protein tyrosine kinase domain which regulates intracellular signal transduction; the transmembrane domain that connects both intra and extracellular domains, the regulatory domain which contains kinase inserts, and sites for auto-phosphorylation [38]. With the exception of IR and insulin-like receptors (ILR), the activation of all RTK takes place *via* the lateral dimerization of their two cytoplasmic catalytic domains as a starting point and the subsequent intermolecular autophosphorylation upon binding to extracellular ligands. The autophosphorylation of particular dimers of RTK in turn activates the signalling cascades to phosphorylate their corresponding cytoplasmic substrates (Figure 2) that regulate their physiological functions through various signalling pathways including the Ras/MAP and PI3 kinase pathways. Some proteins that interact with activated RTK function as adaptor proteins and lack intrinsic enzymatic activity of their own [39–41]. However, under various circumstances RTK may be activated by cytoplasmic proteins, such as cytohesin, which activates EGFR while inhibition of cytohesin signalling downregulates proliferation of EGFR-dependent cancer cells [42]. Activated RTK can initiate “positive and/or negative” signalling mechanisms to regulate a variety of physiological functions. “Positive signals” are involved in stimulation of cells and induction of a variety of responses that includes cell proliferation and differentiation [43]. On the other hand, “negative signalling” decreases the levels of positive signals and modulates cell stimulation levels and therefore acts as a regulator of cellular processes [43]. Understanding the mechanisms underlying RTK activation and their regulation is therefore essential in developing strategies for therapeutic interventions.

## 4. Epidermal Growth Factor Receptors (EGFRs)

EGFR was the first family member of the RTK family to be discovered and comprises of four homologous receptors such as ErbB-1 (EGFR itself), ErbB-2 (HER2), ERbB-3 (HER3), and ErbB-4 (HER4). They all elicit intracellular signalling pathways *via* their agonists including epidermal growth factor (EGF), transforming growth factor-*α* (TGF-*α*), and amphiregulin, to regulate a wide range of biological functions. These include cell differentiation, development, proliferation, angiogenesis, and survival of fibroblasts and epithelial cells [44–46]. Recently, EGFR-mediated signalling pathways have been implicated in various neuromodulatory effects on several types of CNS neurons. These include hippocampal neurons, retinal ganglion cells (RGC) after CNS injury, and in the development of neurological disorders such as Alzheimer's disease [47–49].

### 4.1. The Role of EGFR on Axon Regeneration in an Optic Nerve (ON) Injury Model

On the basis of several studies, the upregulation and transactivation of EGFR elicits both disinhibited axon regeneration of transected ON axons and survival of RGC in chronic glaucoma models [18, 51]. Although, the absolute signalling mechanisms underlying injured ON axon growth inhibition are not well understood, several studies indicate that axon regeneration is at least partially compromised by the activation of EGFR-mediated signalling pathways in association with myelin-derived inhibitory cues. More recently, it has been shown that the pharmacological inhibition of EGFR by local administration of irreversible PD168393 (4-[3(bromophenyl)-amino]-6-acrylamidoquinazoline) and reversible AG1478 4-(3-chloro-anilino)-6,7-dimethoxyquinazoline) EGFR kinase inhibitors to ON lesion sites enhanced adult RGC axon regeneration [52–54].

 Koprivica et al. [52] proposed that EGFR elicits axon growth inhibitory signals in neurons upon the suppression of phosphorylated EGFR. They further suggested that CNS myelin-derived axon growth inhibitory ligands and CSPG stimulate the phosphorylation of tyrosine kinase catalytic domain in a calcium-dependent manner and thereby activate the Rho/ROCK pathway to arrest axon growth. EGFR inhibitors, therefore, counteract the activation of kinase function and block the activities of both myelin- and glial-derived axon growth inhibitory ligands, inhibiting neurite outgrowth. However, the study did not clearly address which particular stimulus is involved, or what molecular changes mediate the Rho/ROCK inhibitory signalling pathway in promoting neurite outgrowth. 

Contrary to this paper, the growth-promoting effects of EGFR antagonists in a similar ON crush model were shown to be mediated by off-target actions of these compounds on resident glial cells in the inhibitory environment. For example, both *in vivo* and *in vitro* observations showed that AG1478 and PD168393 promoted neurite outgrowth of RGC by off-target effects on glia, independently of EGFR [18, 53, 54]. The prerequisite for the direct on-targets effect of these EGFR inhibitors is the expression of phosphorylated (p) EGFR within axotomized RGC somata and on their corresponding axons. But in this *in vivo* paradigm, pEGFR was absent in axons and their corresponding RGC somata while the majority of pEGFR was expressed in ON glia (astrocytes and oligodendrocytes) [53]. On the basis of relevant *in vitro* studies, these EGFR antagonists caused the release of RGC/glia-derived neurotrophins together with elevated cyclic adenosine monophosphate levels (cAMP). This in turn reduced the levels of pEGFR, enhanced the production of mobilizing axogenic proteins, and promoted regulated intramembranous proteolysis of p75^NTR^, thereby inactivating the Rho/ROCK pathway to disinhibit neurite outgrowth in axotomized RGC [53, 54]. Although the mechanism of axon growth promotion by EGFR inhibitors appears to be glial-dependent, they may yet have therapeutic potential in strategies to promote axonal regeneration in the CNS.

Interestingly, EGFR-mediated signalling pathways are implicated in cell growth and proliferation in certain nonneuronal cells such as reactive microglia/macrophages and astrocytes in the CNS [47]. While the activation of the EGFR pathway in the developing CNS enhances the formation of the cribriform structure and provides a supportive environment for neurons and growing axons, this pathway is absent from astrocytes in the adult CNS, but is highly upregulated and activated after neuronal injury. Hence, reexpression and activation of the EGFR pathway under pathological conditions may promote the positive roles of reactive astrocytes in developmental process in the CNS [55, 56]. More recently, heparin-binding epidermal-like growth factor (HB-EGF) and EGFR/MAPK mediated signalling pathways have been implicated in Muller cell dedifferentiation. This is important in the formation of a cycling population of multipotent progenitors to promote retinal regeneration in Zebrafish [57], demonstrating the strategy underlying the inductions of retinal regeneration in certain mammals. 

All these studies have postulated contradictory reasons for the disinhibited axon regeneration upon suppression of EGFR activity and the resulting astrocyte activation in the ON injury paradigm. Indeed, the understanding of the molecular mechanisms behind pharmacological inhibition of EGFR activity and its effects on the inhibitory signalling cascade in axon regeneration is incomplete. However, these observations argue strongly that injury-induced inhibition of ON regeneration is not going to be explained solely by the inhibition of EGFR but rather other combinatorial factors and signalling pathways need to be considered in the context of future therapeutic strategies in injured ON axon regeneration.

### 4.2. The Role of EGFR on Axon Regeneration in Brain and Spinal Cord Injury Models

In most cases, injury to the brain and spinal cord causes major motor and sensory deficits resulting from Wallerian degeneration followed by abortive axonal sprouting and eventual neuronal loss. A convergence of evidence has indicated that inhibition of EGFR can be both neuroprotective and axon growth stimulatory by the modification of astrocyte-mediated inhibitory responses after spinal cord injuries [49, 58]. Although the precise mechanisms underlying the role of EGFR in axon regeneration is not well defined, it has been postulated that transactivation of EGFR is a common critical phenomenon downstream of both intracellular calcium influx and the Nogo-66 receptor signalling activated by myelin- and glial-derived inhibitory ligands [52]. Therefore, inactivation of EGFR may be a potential therapeutic strategy to promote axon regeneration after spinal cord injury [52, 59].

Interestingly, intrathecal administration of the EGFR inhibitor PD168393 enhanced robust regeneration of 5-HT-immunoreactive and Th-immunoreactive axons in the spinal cord accompanied by recovery of hind limb and bladder function in addition to improved sensory function after contusion spinal cord injury in rats. [49]. This study postulated that both the structural and functional recovery observed in these animals resulted from the formation of a permissive growth environment promoted by inactivation of EGFR and astrocytes. This prevented glial scar formation and the secretion of growth inhibitory molecules, such as CSPG. In support of these observations, Han et al. [60], demonstrated that collagen scaffolds containing an EGFR neutralizing monoclonal antibody (151-IgG) together with brain-derived neurotrophic factor (BDNF) fused with a collagen binding domain (CBD-BDNF) and implanted into the lesion site, promoted significant axon regeneration in a rat spinal cord injury model, suggesting the usefulness of EGFR inactivation in this model. 

A contradictory finding has pointed out that activation of EGFR by overexpression of transforming growth factor-*α* (TGF-*α*), a known ligand of EGFR, at the lesion site, enhanced axon outgrowth at the rostral lesion border after spinal cord injury. The presence of an astrocyte-mediated growth permissive environment following upregulation of TGF*α* raises the interesting issue that EGFR-TNF*α* mediated signalling triggers proliferation and migration of astrocytes but intrinsically modulates its state to a functional growth-supportive phenotype that facilitates robust axonal outgrowth after spinal cord injury [61]. Although the expression of EGFR has been detected in a majority of neurons, astrocytes, microglia, and macrophages in the developing and adult brain of mammals, after injury, EGFR localisation predominates in reactive glia [62]. However, EGFR-mediated signalling is not only important for development of the CNS but also to maintain its integrity by regulating functions including neurogenesis and neuronal migration [63–66]. Based on all these studies, there is a conflicting perspective on the role of EGFR and astrocytes, particularly in axon regeneration. Although it is obvious that EGFR has potential roles in the CNS, further studies are required for greater understanding of the EGFR-mediated cascade of events after CNS injury. 

## 5. Tropomyosin Receptor-Kinase Receptors (Trk Receptors)

Neurotrophins, members of the nerve growth factor (NGF) family mediate physiological activities through their specific receptors expressed on target tissues or cells. Depending on the binding affinity of neurotrophins, their corresponding receptors are classified into high affinity RTK and the low affinity pan-neurotrophin glycoprotein receptor p75^NTR^, which belong to the tumour necrosis factor (TNF) receptor family [67–69]. The neurotrophin family itself comprises four different molecules: NGF, BDNF, neurotrophin (NT)-3, and NT-4 (also known as NT-5). These neurotrophins exert their effects through different classes of competent receptors, TrkA, TrkB, or TrkC having different ligand binding specificities. NGF preferentially binds to TrkA, BDNF, and NT-4/5 interacts with TrkB, whilst NT-3 seems more specific to TrkC, but can interact with both TrkA and TrkB receptors [7, 67, 70]. Trk receptors not only share approximately 85% sequence homology in their kinase domain but also have considerable resemblance (50% homology) in their extracellular domain. Therefore, similar sequence homology is found between their extracellular domains that bind their specific ligands, NGF, BDNF and NT-3. Thus, distinct activities of different neurotrophins are observed in the nervous system depending on their differential *in vivo* expression and the level and localization of their corresponding receptors. Although their higher order structural features enable them to bind the same receptors, they share several features in their signalling [21]. Surprisingly, the sequence homology of Trk receptors is closely related to the insulin receptor and both insulin and NGF have relatively similar functions [71, 72]. The level of expression of p75^NTR^ is relatively higher than that of Trk receptors and p75^NTR^ is capable of interacting with all neurotrophins to trigger or inhibit Trk signalling, regulating cell survival and in some instances death of both neurons and glia through co-operation with other Trk receptors [7, 69, 73, 74].

During development, activation of Trk-NGF signalling regulates nonmitogenic effects including differentiation, proliferation, and survival of certain neuronal populations in the nervous system [21]. There is a great deal of interest in Trk family receptors in that they alone can mediate a range of physiological functions in response to the variety of corresponding receptor ligands upregulated after CNS injuries [10, 38, 67]. The expression of NGF, BDNF, NT-3, and NT-4 has been detected in both neuronal somata and their axons in a wide variety of neurons including those in the cerebrum, cerebellum, and hippocampus [75]. However, they have different capacities in promoting relevant physiological functions. For example, BDNF, NT-4/5, and the TrkB-mediated signalling pathway shows the strongest axon outgrowth responses compared to NT-3 and NGF [76]. How neurotrophins regulate these diverse biological effects in development and throughout life in the CNS is under intense investigation. Two possible mechanisms exist: (1), a differential combination with adaptor proteins that then signal through a variety of signalling pathways, such as the Ras/MAP kinase, PI3 kinase, and phospholipase C pathways; and (2), based on temporal expression patterns and spatial location of stimulation, they may elicit signals through their alternative spliced forms [69, 73, 77]. These are functionally distinct splice variants exhibiting either dominant negative or positive functions by their original signalling pathways.

### 5.1. The Influence of Trk Receptors on CNS Axon Regeneration

**Figure 3:**
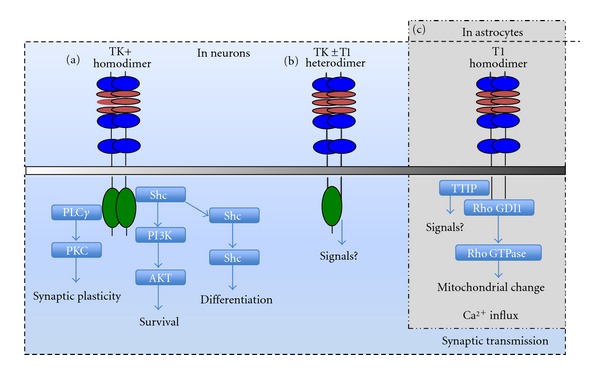
Schematic diagram summarizing the known possible TrkB signalling pathways associated with neuronal plasticity. Within neurons (dotted square), BDNF can induce three types of Trk B dimers to form: (1) TK+ homodimer; (2) TK ± T1 heterodimer and (3) T1 homodimer. The TK+ homodimer results in activation of PLC*γ* and PKC, which promote synaptic plasticity. Shc proteins activate the PI3K-Akt pathway and cell survival while Ras-MAPK pathway activation regulates differentiation. The function of the TK ± T1 heterodimer remains unknown. The T1 homodimer is important in synaptic transmission within neurons but a mechanism for this phenomena has not yet been elucidated. However, T1 is the major isoform of TrkB receptors in astrocytes (grey box), which induces release of Rho GDI1 and is involved in a Ca^2+^ influx in astrocytes. A TTIP (truncated TrkB-interacting protein) binds T1 but its functions are not yet clear. Adapted from [69].

Emerging evidence indicate that neurotrophins and their corresponding receptors play pivotal roles in overcoming the problems of neuronal survival, axon elongation, expression of key enzymes for neurotransmitter synthesis, and remyelination. However, the presence of a permissive environment together with growth promoting cells and/or the use of axonal bridging matrices in the lesion site is a prerequisite for neurotrophins to mediate successful axonal growth in the CNS [2, 9, 78]. Among all the neurotrophins, BDNF and its receptor TrkB are highly expressed in the CNS and TrkB signalling regulates a variety of functions to maintain neuronal plasticity during development through adulthood (Figure 3) [69]. Compared with other receptors, the BDNF-TrkB signalling receptor has been implicated in neuronal survival and axon regeneration in the CNS after injury and in disorders of the nervous system. Whereas BDNF and TrkB signalling pathway promotes RGC survival, a combined intravitreal injection of Forskolin (an elevator of intracellular cyclic AMP levels) BDNF, and CNTF was shown to promote both survival and regeneration of axotomized type-*β* RGCs in cat retina [79]. 

The major obstacles for systemic application of neurotrophins are the blood brain barrier (BBB) and the truncated TrkB receptor on astrocytes in devising treatments for CNS neuropathies and injuries. Exogenous application of BDNF and preconditioning lesions (where dorsal column injury is preceded by preconditioning sciatic nerve lesions) enhance regeneration of ascending sensory axons but also enhanced functional recovery together with expression of p-CREB, pERK, and GAP-43 after spinal cord injury, indicating that BDNF is anterogradely transported by axons [80]. However, the extracellular signal-regulated kinases (ERK1/2) mediated survival but not axon regeneration of adult injured CNS neurons [81]. The combined application of BDNF and NT-3 was reported to promote propriospinal axon regeneration and enhance regeneration of the axons of specific distant populations of brain stem neurons into Schwann cell grafts [82]. By contrast, endogenous NT-3 enhances the death of mature BDNF-dependent axotomized corticospinal neurons antagonistically *via *p75^NTR^ mediated cosignalling *in vivo*. TrkB and TrkC are expressed in unlesioned and lesioned corticospinal neurons while relatively low levels of p75^NTR^ expression is evident after injury, suggesting that the potency of each receptor to elicit their function is determined at the level of their expression in relevant cells [83].

 While endogenous NT-3 promotes death of axotomized spinal neurons, non-physiological upregulation of NT-3 suppresses the expression of TrkC receptors and promotes survival of these neurons, by mimicking BDNF function through TrkB receptors and/or upregulating the expression of BDNF [83–86]. The role of BDNF and its TrkB receptor in promoting survival and regeneration has also been studied in chronically injured rubrospinal tract neurons. It was shown that administration of BDNF to cell bodies in the midbrain or implantation of peripheral nerve tissues at the site of spinal cord injury, significantly promoted axon regeneration together with increased gene expression of GAP-43 and T*α*1-tubulin [87]. In a later study, Kwon et al., [88] reported that rubrospinal tract neurons failed to respond to BDNF when applied to the spinal cord injury site, 2 months after cervical axotomy. This effect was not related to the dose of BDNF administered and indicated that failure of regeneration is not associated with the dose of BDNF administered, but rather spontaneous loss of trkB receptors on the injured axons over time [88]. 

Truncated TrkB, a spliced form of TrkB lacking the catalytic tyrosine kinase domain, plays an important role in BDNF-mediated neurogenesis and synaptogenesis, exhibiting differential responsiveness for axon regeneration after axotomy. For example, upregulation of truncated TrkB and p75^NTR^ appears to inhibit BDNF-mediated neurite outgrowth from CNS neurons after injury [76, 89, 90]. Fryer et al. [76] further reported that the truncated TrkB on non-neuronal cells reduced the availability of TrkB ligands such as BDNF and NT4/5, by selectively removing them from the environment of growing axons, thus inhibiting BDNF and NT4/5-induced neurite outgrowth. However, truncated TrkB-mediated neurite outgrowth is considered a developmental change for synaptogenesis. Upon activation, truncated TrkB on glial cells recruit additional truncated TrkB^+^ neighbouring cells, removing excess BDNF and promoting axonal pruning *via* a dominant negative effect and inhibiting the activity of normal TrkB receptors in neurons [69]. Paradoxically, BDNF and the truncated form of the TrkB receptor play an important role in promoting dopaminergic functional axon regeneration within the nigrostriatal dopamine system after injury to the striatum [91]. The local administration of NT-3 after injury failed to rescue dorsal column sensory axons, which form ascending projections through the dorsal column to the gracile and cuneate nuclei in the medulla, due to the absence of TrkC receptors. Hence, this study emphasises that when developing a strategy for exogenous application of neurotrophins for neuroprotection in spinal or brain injuries, it is important to determine which type of neurotrophic factor receptor exists and the most suitable ligand to promote neuron survival and axon regeneration in the CNS region of interest. To support this notion, another study also reports that local administration of NT-3 failed to rescue dorsal column sensory axons after spinal cord contusion injury, since these axons lacked TrkC receptors [92]. Based on all these studies, the functions of Trk receptors in the regulation of axotomized CNS neurons, their survival, and regeneration are crucial after traumatic injury. 

**Figure 4:**
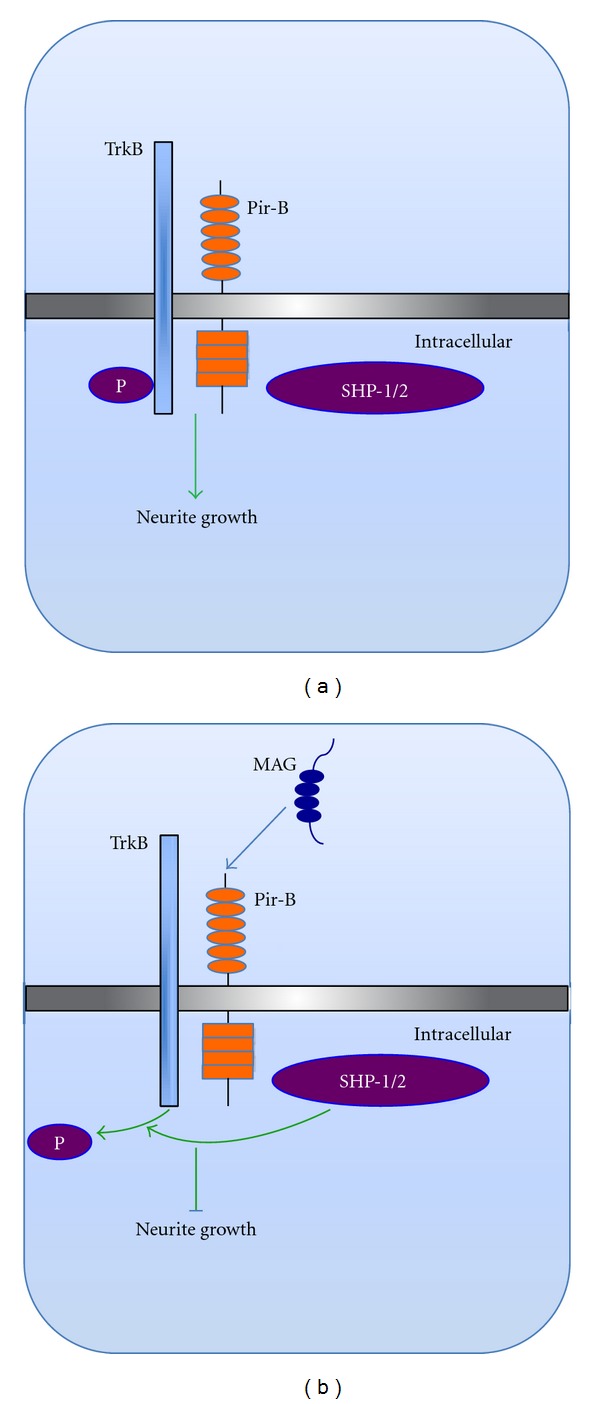
Molecular pathway of PIR-B signal transduction. Binding of appropriate ligands to PirB leads to formation of a receptor complex along with TrkB which then recruits SHP1/2 and their interaction deactivated TrkB and therefore neurite outgrowth is inhibited. Adapted from [108].

The importance of paired immunoglobulin-like receptor B (PIR-B)-mediated inhibition of TrkB activity and its role in axon regeneration has recently been reported. PIR-B is considered as a high affinity receptor for myelin-derived axon growth inhibitory ligands such as Nogo, MAG, and OMgp [93] and inhibition of PIR-B-mediated signalling pathways enhanced axon regeneration of axotomized RGC *in vivo* [94]. This study further postulated that a state of imbalance between the expression of TrkB and PIR-B modulate the sustainable axon regeneration of injured axons. Upon downregulation of TrkB, ligands such as BDNF and NT-4/5 and the inhibitory molecules in the CNS that bind to PIR-B form a receptor complex with TrkB and the consequent recruitment of Src homology 2-containing protein tyrosine phosphatase (SHP-1/2). This in turn induces dephosphorylation of the kinase catalytic domain of TrkB and blocks axon regeneration (Figure 4). In the presence of an axon growth inhibitory environment, agents that either activate TrkB or inhibit the binding of BDNF to p75^NTR^ can be used as better therapeutic candidates in CNS axon regeneration since the BDNF/  p75^NTR^ complex potentially compromises the ability of BDNF to activate TrkB to enhance growth of axotomized CNS axons [95]. On the basis of these studies and despite the extensive use of neurotrophins, the family of Trk receptors raises challenging issues in terms of signalling cascades and their regulatory mechanisms in axon regeneration in the CNS. There are many issues, which remain to be explored, and obstacles to be tackled in the *in vivo* uses of neurotrophins as therapeutics in the treatment of CNS injuries. These issues will be briefly discussed later in this paper.

Even though both Trk receptors and EGFR have been extensively studied and subjected to therapeutic approaches in the CNS and particularly after spinal cord and ON injury, other growth factors such as insulin-like growth factor- receptor (IGF-1R), fibroblast growth factor receptor (FGFR), ciliary neurotrophic factor receptor (CNTFR), and glial cell-derived neurotrophic factor (GDNFR) remain of considerable potential targets in CNS injury. More recently EphA4 receptor, also a subfamily of RTK has been implicated in CNS injury. The inhibition of EphA4 promotes axon regeneration and functional recovery by blocking astrocyte gliosis in spinal cord injury models and this receptor type may be subjected to considerable attention in future studies for the treatment of spinal cord injuries [96].

## 6. GDNFR

GDNF is a family member of the transforming growth factor-beta (TGF-*β*) superfamily and preferentially binds glycosylphosphatidylinositol (GPI)-anchored protein receptor (GFR), which is dynamically located on the plasma membrane. The GDNF receptor family comprises of GFR*α*1, GFR*α*2, GFR*α*3, and GFR*α*4. Among them, GFR*α*2 is highly expressed in the cortex, basal forebrain, specific layers of the olfactory bulb, cerebellum and motor nuclei [97, 98]. The alternatively spliced isoforms of GFR*α*2, such as GFR*α*2a and GFR*α*2c, also promote cAMP-mediated axon growth [99]. Initially GDNF was thought to play a survival-promoting role in CNS neurons, since it enhanced the survival of injured dopaminergic neurons, motor neurons, and RGC [79, 100, 101]. It has been suggested that GDNF activates Muller cells to secrete growth factors and thereby promotes the survival of axotomized RGC [79].

GDNF is generally considered a potent neurotrophic factor for motor neurons and enhances their survival and axon regeneration after implantation of peripheral nerve grafts in spinal root avulsion models [102]. *Ex vivo* gene delivery of GDNF has been shown to promote extensive growth of motor and dorsal column sensory axons and remyelination of regenerating axons by recruiting more Schwann cells to the lesion site after partial and complete spinal cord transections [103]. GDNF-mediated Schwann cell migration and myelination is regulated by cAMP-dependent protein kinase A and protein kinase C signalling pathways [104]. The survival-promoting effects of GDNF on spinal motor neurons are restricted to fusimotor subtypes [105] and disruption of specific GDNFR subtype signalling compromised cortical neuronal survival in Alzheimer's brains [106]. GDNF-enriched acellular nerve grafts enhanced motor neuron axon regeneration after implantation into cervical root avulsed-spinal cords [107]. These studies highlight the importance of GDNFR, both in survival and in axon growth of different CNS neuronal subtypes.

## 7. CNTFR 

CNTF, a potent survival factor for neurons and oligodendrocytes, elicits its signals after binding its receptor, CNTFR, promoting neurotransmitter synthesis, neuronal survival, and neurite outgrowth in certain neuronal populations. Previous studies reported that CNTF stimulates neurite outgrowth from spinal cord neurons and the neurite growth-promoting effects of CNTF, however, does not appear to be a consequence of its survival-promoting effect [109]. Unlike NGF, CNTF also supports survival and/or neurite outgrowth of many neuronal cell types but not synapse formation in adult Lymnaea neurons [110].

The axon growth promoting and neuroprotective effects of CNTF have been extensively studied in injured RGC and accumulating evidence suggests that CNTF profoundly enhances both survival and axon regeneration through activation of the JAK/STAT3 and PI3K/Akt pathways [111–113]. Generally, CNTF expression is strongly unregulated in astrocytes in the retina compared to other nonneuronal cells. The upregulation of CNTF by activation of Muller cells and astrocytes are key mediators that promote RGC axon regeneration and therefore, CNTF appears to switch mature RGC into a regenerative growth activated state after inflammation-mediated axon growth stimulation [112]. ON axotomy and crush accelerates the rapid loss of CNTFR from the proximal and distal stumps of injured axons and consequently limits the ability of RGCs to respond to CNTF and ultimately undergo apoptosis. On the other hand, CNTFR on astrocytes and Muller cells activate them to promote scarring as well as enhance the secretion of other neurotrophic factors, such as fibroblasts growth factor (FGF) [114–116]. A recent study indicated that AAV-mediated delivery of CNTF gene combined with short-term pharmacotherapy at the time of, or just after ON injury, is neuroprotective and enhances RGC axon regeneration and may be a potential treatment in acute CNS injury [111, 117].

### 7.1. IGFR-1

The effects of IGF-1 have also been studied in various neuronal populations. In one study, IGF-I gene delivery enhanced adult corticospinal neuron survival but failed to promote their axon regeneration after injury [118]. In addition, IGF-I has been implicated in RGC survival [119], while BDNF and IGFR-1 enhanced chick bulbospinal neurite outgrowth *in vitro* [120]. IGF-1 also plays pivotal roles in oligodendrocyte development, survival, and myelin synthesis [121] IGF-1 mediated activation of a novel type of nonastrocytic inner retinal glia-like (NIRG) cell exacerbated damage to neurons and Muller cells. This suggests that IGF-1 may also have a negative impact on the survival and regeneration of injured RGC axons [122]. In contrast, a study using a goldfish model reported that early upregulation of IGF-1 after ON injury is a prerequisite for RGC survival and axon regeneration in the adult through activation of the PI3K/Akt signalling pathway [123]. To support this study, IGF-1 mediated significant levels of RGC survival and axonal growth in a rat ON crush model after trans-corneal electrical stimulation of the retina [102]. Based on these observations, IGF-1 appears to have a potential role in axon regeneration but its activity is time-dependent in ON injury models.

## 8. Reasons for the Failure of Trk Receptor-Mediated Survival and Axon Regeneration

### 8.1. Differential Neuronal Cell Body Responses

In order to obtain complete functional regeneration, surviving injured adult neurons must respond either by reexpressing growth associated molecules, respond to exogenously applied neurotrophins, or respond to other appropriate stimuli to trigger robust axon regrowth. In contrast to the PNS, there is a differential cell body response after injury in the CNS. Regardless of injury sites being either proximal or distal to the cell body, axotomized motor neurons regenerate their axons into PN grafts, while graft-induced regeneration of rubrospinal axons only occurs after axotomy proximal to their cell bodies, indicating that appropriate cell body responses to injury are a prerequisite for CNS axon regeneration [22]. Another study has pointed out the importance of early tissue priming and exact orchestration of different steps to different temporal expression patterns of growth factors in white and grey matter. For example, GDNF, CNTF, hepatocyte growth factor (HGF), FGF-2, and BDNF were elevated in the corpus callosum but not in the cortex, indicating that tissue differences in the molecular regulation of remyelination in the white and grey matter exists [124].

### 8.2. Combinatorial Effects

It is well documented that both PNS and CNS neurons require trophic factor-derived signals for their survival and regeneration [9, 10, 125–127]. The CNS requires a combination of molecules for trophic support of its different neuronal populations compared to PNS neurons, reflecting the greater complexity of collaborative signalling pathways that exist in the CNS. Thus, a single neurotrophin is insufficient to shut down the pathway to apoptosis and overcome the repulsive barrier molecules required for neurons to regenerate their axons [128–131]. However, a combination of intravitreally injected neurotrophic factors such as BDNF, CNTF, FGF2, GDNF, NT3, NT4, and raising the levels of cAMP, effectively enhanced both RGC survival and axon regeneration in ON transected animals [79, 132–135]. The requirement for combined trophic stimulation may be due to the connection of certain neurons to multiple target tissues and their different receptors, suggesting that multiple RTK families and other receptors need a collaborative response to effect maximum neuronal survival and axon regeneration. 

### 8.3. Availability of NGFR in Growing Axons

The ability of particular neurotrophic factors to promote survival and axon regeneration depend on the localization of their receptors in specified locations that are then retrogradely transported to the cell body *via* their axons [136]. For example, grafting BDNF-secreting cells promote axon growth of injured rubrospinal neurons but not the growth of corticospinal axons because of the expression of TrkB on the rubrospinal projecting axons [137]. Therefore, to design a strategy to promote survival and axon regeneration, we first need to clarify the specific contribution or effects of each trophic factor and their receptor locations in particular neurons of interest. Inadequate information about independent effects of each ligand on CNS neuronal subtype currently exists and need further investigation.

### 8.4. Delayed Repair and Slow Axonal Growth

Numerous experimental strategies to promote axon regeneration have shown their feasibility in animal models of acute spinal cord injury, but their effectiveness often declines with a delay in administration of neurotrophic factors. Appropriate timing is therefore crucial for the repair of injuries and their potential for axon regeneration and restoration of lost function. Once sprouting injured axons have encountered growth-inhibiting cues in the external environment, they lose their ability to respond to growth promoting factors as well as other intracellular growth mediators, including signals derived from RTK [2, 138, 139].

### 8.5. Mode of Delivery of Growth Promoting Factors and Their Receptors

Several experimental strategies have been employed to minimize tissue damage during the exogenous application of neurotrophic factors and to enhance axon growth and functional restoration after CNS injury. The mode of administration of growth factors has been under scrutiny since some methods downregulate the effectiveness as a result of damage around neuronal tissues, altering morphological as well as functional recovery. Currently, there are several fascinating techniques, which exist, including implantation of appropriate cells and tissues at the site of injury, gene therapy, and electrical stimulation. Among them, gene therapy is the most popular method to deliver neurotrophic factors and holds the most therapeutic promise [23, 140].

## 9. Conclusion

In summary, there are now several experimental strategies that have been established to promote neuronal survival and regeneration of their axons to improve function after CNS injury. Among these approaches, upregulation of neurotrophic factors and their receptors, many of which belong to the RTK family, have a great impact on CNS axon regeneration. These strategies target many of the cellular processes behind axon regeneration such as demyelination, axon retraction, sprouting, and neuronal death. Thus far, combinatorial signalling pathways that mediate axon regeneration can be targeted for sufficient functional impact. However, simultaneous and spontaneous administration of cAMP and neurotrophins are more effective in promoting axon regeneration in CNS injury models. Faced with the significant barriers to axon regeneration in the CNS, it may be that only a modest level of axon regeneration is sufficient to promote substantial functional restoration—a feat, which is probably achievable in the not too distant future. 

Despite extensive studies and encouraging data on a variety of neurotrophins and their roles in promoting survival of injured adult neurons and regeneration of their axons, many challenges yet remain for RTK and their ligands prior to their therapeutic use in humans. The inherent complexity of the adult CNS, the plethora of RTK and their ligands as well as their different roles apart from neuronal survival and axonal regeneration make the therapeutic approaches more complex. Therefore, many issues need to be resolved before use in CNS regeneration, stroke, and other neurological disorders including Parkinson's and Alzheimer's diseases. Hence, a combinatorial approach is the most promising strategy to enhance the ability of injured axons to promote CNS axon regeneration.
